# Triple Burden of Obesity, Undernutrition, and Cardiovascular Disease Risk among Indian Tribes

**DOI:** 10.1371/journal.pone.0147934

**Published:** 2016-01-25

**Authors:** Gautam K. Kshatriya, Subhendu K. Acharya

**Affiliations:** Department of Anthropology, University of Delhi, Delhi-110007, India; INIA, SPAIN

## Abstract

**Background:**

Socio-cultural transitions among individuals from vulnerable groups introduce epidemiological transition, with a concomitant increase in the prevalence of undernutrition, obesity, and cardiovascular disease risks. An accepted conventional wisdom exists for Indian tribes that they are undernourished and away from lifestyle-related diseases. However, the extent of this triple burden affecting them is unknown. In this study, we assessed this triple burden among the 9 major tribes of India.

**Methods and Findings:**

During January 2011 to December 2013, we conducted a cross-sectional study among 1066 men and 1090 women constituting a total of 2156 adults belonging to the 9 major tribal groups: Santals, Oraons, and Koras (West Bengal); Santals, Bhumijs, and Bathudis (Odisha); and Dhodias, Kuknas, and Chaudharis (Gujarat) to estimate the prevalence of the triple burden (undernutrition, overweight or obesity, and hypertension). A high prevalence of undernutrition and hypertension was observed among the Koras (51.9%and 10.6%, respectively), Bathudis (51.3% and 12.1%, respectively), and Oraons (49.6% and 16.5%, respectively). However, the prevalence of overweight and hypertension among the Bhumijs (17.7% and 14.7%, respectively), Dhodias (23.8% and 12.9%, respectively), Kuknas (15.8% and 11.3%, respectively), and Santals of West Bengal (12.2% and 11.8%, respectively) and Odisha (15% and 9.6%, respectively) was most alarming. The prevalence of overweight or obesity among the women was 10.9% and 1.5%, respectively, with 14.0% hypertensive women. The prevalence of overweight and obesity among the men was 14.8% and 1.7%, respectively, with 9.2% hypertensive men. Undernutrition was highly prevalent among men and women. However, data from the past 30 years on systolic blood pressure (SBP) and body mass index (BMI) revealed that the studied tribes were at a higher risk than the general Indian population. In addition, a vast gender disparity with relation to the disease and risk prevalence was observed.

**Conclusion:**

The alarming trend of an increasing prevalence of overweight/obesity, undernutrition, and hypertension is observed among indigenous populations of India, emphasizing the incorporation of a specific health management policy.

## Introduction

Indian tribal populations are experiencing phenomenal changes on the social, cultural, and economic fronts, for the past 50 years [1]. Like all developing countries, large-scale developmental activities and urbanization in India have brought significant changes in the lifestyles, occupational patterns, and dietary habits of these tribal communities, once considered outreach groups. Furthermore, new "urban centers" are developing quickly near rural and tribal areas [1–3]. As the health issues of tribal infants and children are increasingly being recognized, few concrete efforts have been made to understand the problems of adult and elderly population with special reference to emerging public health problems of degenerative diseases, such as diabetes, obesity, and cardiovascular diseases (CVDs) [1].

Obesity is a complex disorder and a major health risk factor linked to CVD, stroke, cancer, hypertension, diabetes, and early death [4]. In his review, Benyshek (2007) revealed that the increasingly high prevalence of obesity, among Pima Indian tribal men and women of southern Arizona, is commonly noted among groups that have experienced severe cultural and economic disruptions with prolonged food insecurity, followed by a rapid transition to more refined foods [5]. Currently, the health profile of Indian tribes is potentially undergoing a similar transition. However, a comprehensive study on the health status of the tribes regarding the life style changes is not available, except for the one conducted by the National Nutrition Monitoring Bureau (NNMB) in 2009. This study reported a 2–3% prevalence of overweight among the Indian tribes. On the contrary, India ranks 135^th^ of the 187 countries in the 2014 UNDP Human Development Index and 55^th^ of the 76 countries in the Global Hunger Index [6].

A considerably large burden of this socio-economic disadvantage is reported among Indian tribal populations. Undernutrition has been a major health concern among India tribal populations [1, 7–16]. The Indian National Family and Health Survey 2005–06 indicated a 47–48% prevalence of undernutrition among the tribes [17]. Previous studies have shown that the mortality and morbidity from chronic diseases among Asian populations is prevalent among people with a low body mass index (BMI), and thus they tend to accumulate intra-abdominal fat without developing the generalized risk of obesity, i.e., BMI>23 kg/m^2^ [18,19]. However, various developmental and economic activities undertaken by the state and central government agencies have allowed several tribes to lead a comparatively affluent lifestyle in varying proportions, which has made them vulnerable to various metabolic risk factors. This apparent shift is converse to the traditional wisdom regarding tribes that they do not encounter lifestyle diseases

Hypertension, a direct indicator of CVD, has been strongly implicated for the increased prevalence of overweight/obesity [20–22] and undernutrition [23, 24]. Hypertension is the third most prevalent risk factor for the disease burden in south Asia [25]. The overall prevalence of hypertension in India was reported as 29.8%, with 27.6% and 33.8% in rural and urban areas, respectively [26]. In their meta-analysis Rizwan et al. (2014) demonstrated a 16.1% prevalence of hypertension among Indian tribes with considerable heterogeneity [27]. In a 2007–08 study, NNMB highlighted that hypertension prevailed among approximately 25% men (17.5% stage I and 7.7% stage II hypertension) and 23% women (15.5% stage I and 7.5% stage II hypertension) [28].

Few large-scale studies have investigated the co-prevalence of undernutrition and hypertension involving multiple Indian tribes, with the last study being conducted in 2007–08 [28]. The present study is one of the recent-large-scale studies which involve nine tribes. Similarly, the status of obesity among the tribes was not reported, except in the study conducted by the NNMB in 2007–08. However, the high prevalence of hypertension among many Indian tribes and the absence of a comprehensive and updated prevalence of undernutrition and obesity along with hypertension make the formulation of any viable policy in this regard extremely difficult. Therefore, conceptualizing such baseline studies on the prevalence of malnutrition and lifestyle-related diseases is essential. The present study estimated the prevalence of the triple burden of obesity, undernutrition, and hypertension and highlighted the risk for potential CVDs among nine major Indian tribes from different geographic locations of three states.

## Material and Methods

### Ethical statement

Prior ethical clearance to conduct the research was obtained from the Institutional Review Committee, Department of Anthropology, University of Delhi. Informed written consent from the participants of the study was obtained prior to the actual commencement of the study.

### Area and people

**Fig 1:**
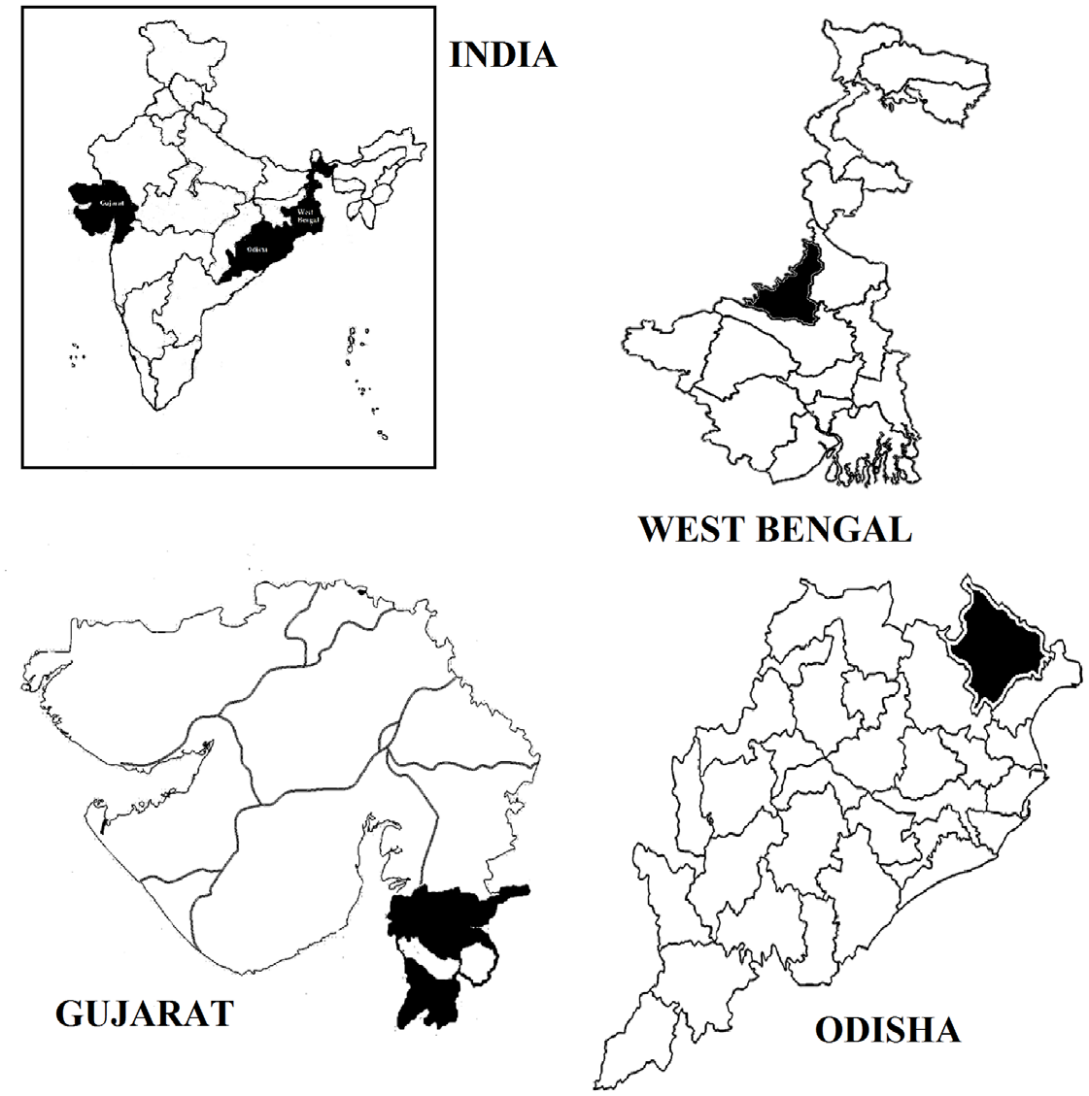
Geographic locations of the selected districts in West Bengal, Odisha and Gujarat on map of India.

The present study included nine tribes belonging to West Bengal (Santal, Oraon, and Kora), Odisha (Bhumij, Santal, and Bathudi), and Gujarat (Dhodia, Kukna, and Chaudhari) (Fig 1). The selected groups are major Indian tribes.

The Indian tribal populations are socially and economically underprivileged groups. Traditionally, the tribes of West Bengal and Odisha mainly earned their livelihood from forest produce, cultivation, and manual labour in farms. However, because of industrial growth and other developmental activities, several tribal people of West Bengal and Odisha are migrating and accepting menial jobs which alter their dietary habits as well. Conversely the tribes of Gujarat are agriculturists and enjoy a relatively sedentary life. They are also involved in government jobs, cattle rearing, and manual labour. The tribes of Gujarat are the most affluent of all the tribes included in the present study.

Although all the studied groups have access to basic amenities such as water, electricity, education, and health care, there are disparities in the availability of these facilities because of social and economic inequities.

Community activities such as traditional folk singing and dancing practices, among the Santals, Dhodias, and Chaudharis, are being replaced by television and video shows. Similarly, modern entertainment equipment, such as television sets and radios are present in most households.

The present study findings reveal that alcohol consumption was higher among the tribes of West Bengal and Odisha, both in men and women. However, alcohol consumption among the tribes of Gujarat was lower than that among the tribes of the other two states.

### Sample

The sample collection was based on a multi-stage sampling method. Three states were selected from two different regions, two from the eastern region (West Bengal and Odisha) and one from the western region (Gujarat) of India. Three tribes were selected from each state based on their predominant distribution. Furthermore, a total of 66 tribal villages from the four districts in three states were chosen on the basis of their residence in the acculturated areas of development. These villages are the original tribal settlements with access to developmental activities. These areas are comparatively closer to the ‘urban centers’ than to the typical countryside habitation. Village listings for each of the tribe were prepared on the basis of their population concentration. We first estimated the number of men and women into four 10-year age interval groups (20–60 years) across several villages with the preponderance of specific tribal inhabitants in the population. A sample size of 30 men and women from each of the four 10-year age interval groups was selected using systematic random sampling.

Therefore, the study comprised a total sample size of 2156 adult tribal participants, with 1066 men and 1090 women (four less than the target sample size).

Exclusion criteria were as follows: growth and developmental disorders, severe health issues in the past year, and the existence of any secondary cause of hypertension. The sample size for the present study was tested at 5% level of significance, with a power of 80%.

### Field survey design

The present cross-sectional study was conducted between January 2011 and December 2013 in five different phases to collect data on selected biomarkers. Each tribe was identified as one cluster, and data from every cluster were collected during the same time.

### Measurement of anthropometric and metabolic variables

The primary information of the participants such as name, tribe name, age, sex, and other family information was recorded in a structured format. Standard techniques were followed while taking all the anthropometric measurements [29]. The standing height and weight was measured to the nearest of 0.1 cm and 0.1 kg respectively. Stature was measured using a movable anthropometer; body fat percentage was measured by bioelectrical impedance analysis (BIA) using Omeron Karada Scan Body Composition Monitor, which is a handheld impedance analyser. Personal particulars such as weight, height, age, and sex of a participant were first recorded in the instrument, followed by the recording of the impedance and body fat percentage calculation. The BIA provides the measurement of the conductance of a small alternating current in the human body [30]. The amount of water in fat-free mass determines the conductance, which allows the determination and assessment of the fat-free mass, and its difference with the body weight gives the body fat percentage [30]. BIA (total body) primarily gives the measurement on impedance from foot-to-hand [31]. The instrument for BIA is easy-to-use, inexpensive, and portable and provides a better accuracy in field circumstances [32]. Care was taken to ensure that each participant received at least 1 hour of rest before the measurement and did not consume any alcohol or plenty of water. Minimum waist circumference was measured using a nonexpendable measuring tape to calculate the waist to height ratio (WHtR). Weight was also measured using the Omeron Karada Scan Body Composition Monitor. The participants were encouraged to remove their shoes and heavy clothing before the measurements. BMI was calculated as weight in kilogram (kg) divided by height in meter squared (m^2^): kg/m^2^. Systolic and diastolic blood pressure (SBP and DBP respectively) were recorded twice using a standard mercury sphygmomanometer on the right arm of the participants. A minimum 15-minute rest before the measurement and a 5-minute interval between two measurements were ensured. The average of the two measurements was recorded.

### Research team and sample collection method

Two trained anthropologists guided by the principal investigator constituted the field research team for the data collection. To avoid measurement and data entry bias, all measurements were taken by one anthropologist, while all data were entered in the datasheet by another. During the study, the same instruments were used for recording the measurements of all the sampled participants. The selected villages were informed before the commencement of the study. Participants who avoided the sampling were excluded.

### Individual classifications

High BMI has been treated as a strong indicator of obesity and CVD in various populations. Furthermore, the "obesity paradox" has been reported which indicated that low BMI was associated with hypertension [33, 34]. Therefore, we considered both low and high BMI statuses as predictors of CVD risks. According to the World Health Organization (WHO) guidelines for Asian populations, individuals with BMI<18.5 kg/m^2^ were considered as underweight; ≥18.5 kg/m^2^ but <23 kg/m^2^as normal; ≥23 kg/m^2^ but <27.5 kg/m^2^ as overweight; ≥27.5 kg/m^2^as obese [35–37]. BMI≥25 kg/m^2^ was also calculated for public knowledge.

Several studies have demonstrated that WHtR is more closely associated with central obesity, encompassing the adjustment to various statures [38–40] and overcoming the negative correlation of height to metabolic syndromes [41]. The mean height of Indian tribes is less than that of the mean height of the general Indian population [42, 43]. Therefore, WHtR can be an ideal obesity measure and a strong indicator of health [44] in Indian tribal populations. In the present study, WHtR was classified in to four categories: (1) <0.40 as underweight;(2) ≥0.40 to <0.50 as normal [45]; (3) ≥0.50 to <0.60 as high risk; and (4) ≥0.60 as morbidly high [46].

Based on the classification given by Muth, 2009 [47], we have subdivided the body fat percentage into four categories, as underweight (10–13% for women; 2–5% for men), normal (14–24% for women; 6–17% for men), at risk or overweight (25–31% for women, 18–24% for men), and morbid/obese (>32% for women; >25% for men).

Participants with blood pressure ≥140/90 mmHg were considered as hypertensive [48].

### Statistical analysis

After incorporating and systematizing the data into Microsoft Excel 2007, further analyses were conducted using SPSS version 16.0 for Windows (SPSS Inc., Chicago, Illinois, USA). The integrity of the sampled data was maintained using double entry. The data were cross-checked several times to ensure its validity and accuracy. Descriptive statistics, such as mean and standard deviation (SD), were used for the selected anthropometric and physiological variables. The prevalence of normal and selected risk categories was calculated for each selected variable in percentages, with a 95% confidence interval (CI). The prevalence percentages of hypertension in men, women, and the overall population were calculated. Undernutrition, overweight, BMI, WHtR, and body fat percentage were estimated and presented in bar graphs. A tribe-wise body fat percentage among men and women in each risk category was presented in a line graph. The percentages were calculated at a 95% CI. The mean and SD for normal and undernutrition states were calculated for men, women, and the overall population in each tribe and as a whole. The patterns of mean SBP and mean BMI among the general Indian population in the past 30 years and those observed among men, and women, and the overall population were compared, and the results were plotted as line graphs.

## Results

### Sample characteristics

Table 1 describes the demographics and characteristics of the study population. The sample was at a shortage of 14 men and an excess of 10 women from the predetermined sample size of 1080 men and women each.

**Table 1:** Demographics and population characteristics of selected tribes.

Name of the tribes	Sex	Sample Size	BMI	WHtR	Body Fat Percentage	SBP
**Santal (West Bengal)**	Males	123	19.9±2.6	0.44±0.05	18.4±5.7	127.5±18.2
	Females	122	19.5±3.2	0.44±0.05	27.3±6.1	123.9±24.0
	Total	245	19.7±2.9	0.45±0.05	NA	125.7±21.3
**Kora**	Males	114	18.9±2.0	0.43±0.04	17.3±6.5	124.9±21.0
	Females	121	17.6±2.9	0.42±0.42	23.5±6.5	124.5±22.3
	Total	235	18.3±2.6	0.43±0.04	NA	124.7±21.6
**Oraon**	Males	112	19.6±2.5	0.43±0.05	17.2±6.5	124.1±15.4
	Females	124	18.1±2.8	0.43±0.04	25.4±6.1	130.9±20.0
	Total	236	18.8±2.8	0.43±0.05	NA	127.7±18.3
**Santal (Odisha)**	Males	121	20.2±2.8	0.46±0.06	18.7±6.2	125.8±16.9
	Females	119	20.3±3.0	0.48±.05	27.7±7.0	125.4±15.1
	Total	240	20.3±2.9	0.47±0.05	NA	125.6±16.0
**Bhumij**	Males	116	20.9±3.1	0.48±0.07	18.8±6.4	128.7±21.4
	Females	122	19.7±2.9	0.46±0.05	27.3±5.8	129.9±25.1
	Total	238	20.3±3.1	0.47±0.05	NA	129.4±23.4
**Bathudi**	Males	119	19.5±2.8	0.48±0.05	15.5±7.2	125.4±17.9
	Females	121	18.0±2.8	0.45±0.04	24.3±6.1	132.8±23.2
	Total	240	18.6±3.3	0.47±0.04	NA	129.1±21.0
**Dhodia**	Males	121	20.5±3.2	0.49±0.08	23.2±5.8	129.0±20.4
	Females	120	20.7±3.5	0.46±0.06	31.4±6.0	129.6±20.4
	Total	240	20.6±3.3	0.48±0.06	NA	129.3±20.4
**Kukna**	Males	120	20.3±3.1	0.49±0.06	21.6±6.9	128.4±17.4
	Females	120	19.9±2.9	0.46±0.05	30.1±5.7	127.1±16.8
	Total	240	20.1±3.0	0.47±0.06	NA	127.8±17.1
**Chaudhari**	Males	120	19.8±3.1	0.48±0.07	22.1±5.7	125.7±23.5
	Females	121	18.9±2.9	0.42±0.05	28.2±6.1	123.7±21.7
	Total	241	19.4±3.0	0.45±0.07	NA	124.7±22.5
**Total**	Male s	1066	20.0±2.9	0.47±0.06	19.2±6.8	126.7±19.3
	Females	1090	19.2±3.1	0.45±0.05	27.2±6.6	127.6±21.4
	Total	2156	19.6±3.1	0.46±0.06	NA	127.1±20.4

NA = Not applicable

#### Population characteristics

**Fig 2:**
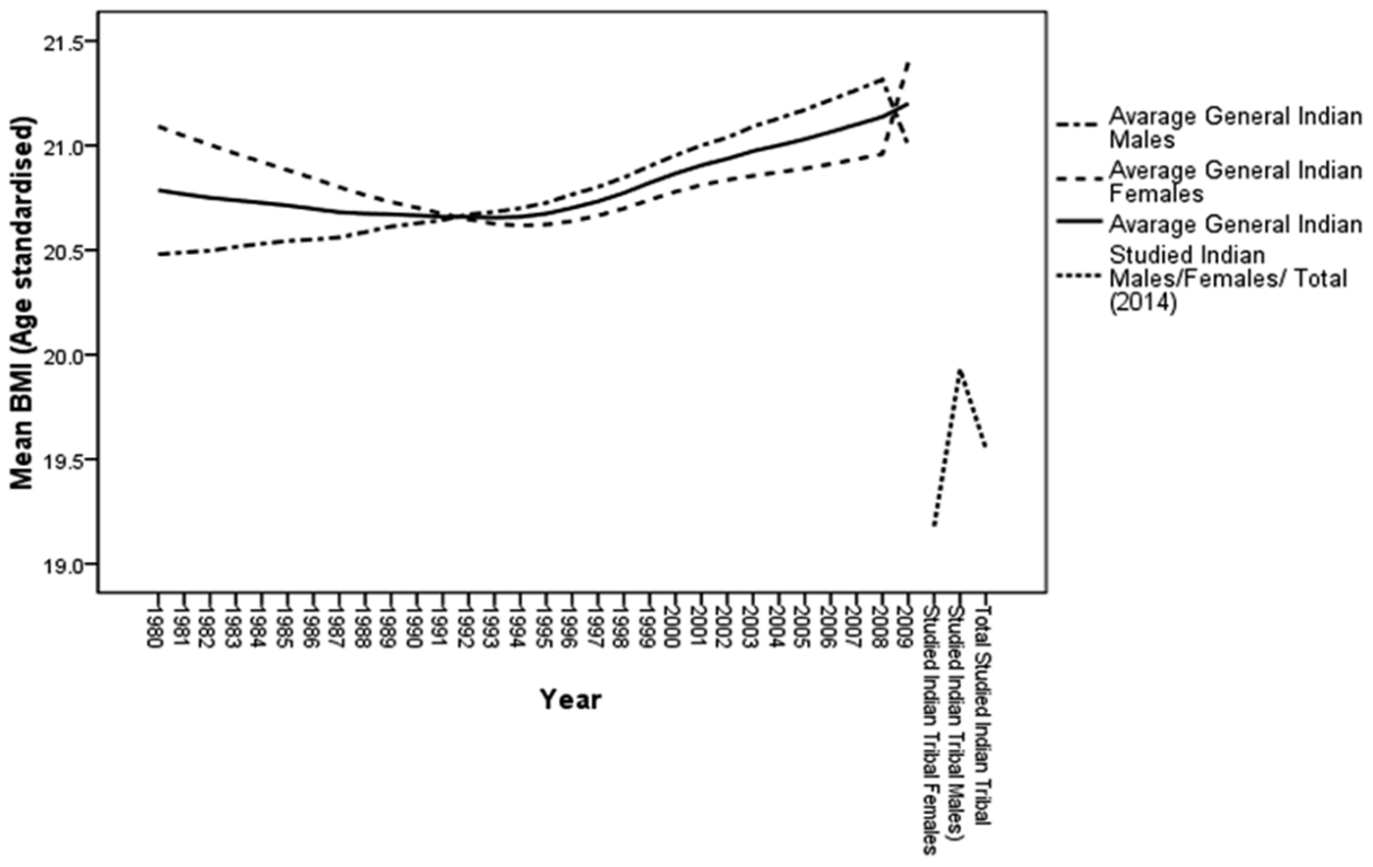
30-year trend of body mass index (BMI) of Indian population compared to its present status among tribal population.

Table 1 shows the population characteristics with respect to selected variables among the studied population groups. A high prevalence of undernutrition was observed among the Koras, Oraons, and Bathudis; women of these tribes displayed alarming mean BMI levels of <18.5 kg/m^2^. Furthermore, the mean BMI among men, and women, and the overall study population was considerably lower than that among the Indian population (Fig 2).

**Fig 3:**
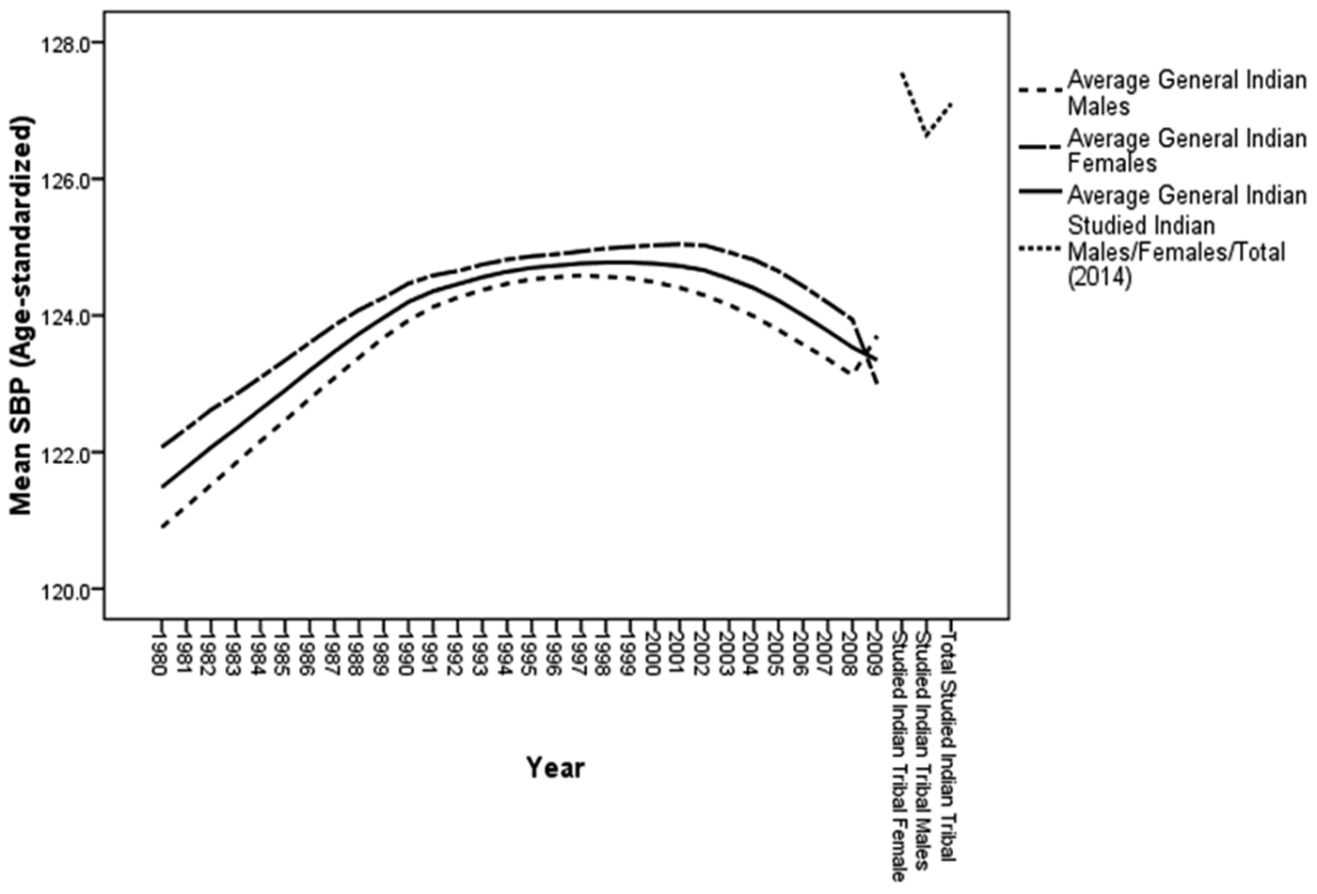
30-year trend of systolic blood pressure (SBP) of general Indian population compared to its present status among the tribal population.

With regards to the mean SBP, the overall status among Oraons and Bathudis was nearly pre-hypertensive while the mean SBP among the women of these tribes was pre-hypertensive (Oraons = 130.9±20.0 mmHg and Bathudi = 132.8±23.2 mmHg). The mean SBP among men, and women and overall study population was considerably higher than that of the average SBP in the general Indian population (Fig 3).

The average WHtR among men and women, and the overall study population was normal for all the nine tribes.

**Fig 4:**
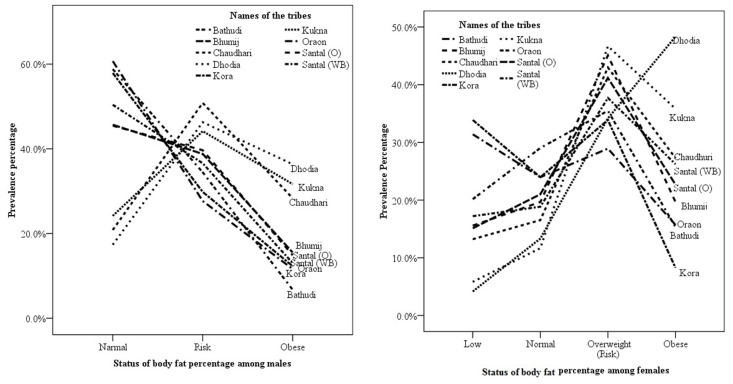
Distribution of body fat percentage under low, avarage, at risk (overweight) and obese categories among men and women of nine tribes.

The mean body fat percentage indicated a high risk among men and women from the three tribes of Gujarat, which are Dhodias (men = 23.2±5.8%; women = 31.4±6.0%), Kuknas (men = 21.6±6.9%; women = 30.1±5.7%), and Chaudharis (men = 22.1±5.7%; women = 28.2±6.1%). Unlike men, women from the nine tribes exhibited a high tendency of a low body fat percentage. Tribes from Gujarat exhibited minimal tendency for a low body fat percentage; whereas tribes from Odisha and West Bengal exhibited a high tendency for a low body fat percentage (Fig 4). A high body fat percentage among men and women of the tribes from Gujarat indicated an increasing trend of fat deposition and a predictable risk of obesity.

The prevalence of hypertension, undernutrition, and overweight and/or obesity among the nine tribes is presented in Table 2. The prevalence of hypertension among the nine tribes was observed to be 11.7% (95% CI 10.5–13.2), that ranged between a minimum of 6.2% (95% CI 3.8–10.0) among the Chaudharis and a maximum of 16.5% (95% CI 12.3–21.8) among the Oraons. Seven of the nine tribes displayed >10% prevalence of hypertension. Furthermore, considering the nutritional status, a high rate of undernutrition was observed. The prevalence of undernutrition varied from as low as 28.8% (95% CI 23.0–34.5) among the Dhodias to >50% among the Koras and Bathudis. In addition, increasing trends of overweight and obesity were observed among the studied tribes. Overall, 12.8% (95% CI 11.5–14.3) of the total participants were overweight and/or obese, with four of the nine tribes exhibiting more than 15% and another two tribes exhibiting more than 10% prevalence. Six of the nine tribes exhibited a little more than 2% prevalence of obesity, with the Dhodias exhibiting the highest prevalence of 2.9% (95% CI 0.01–0.1).

**Table 2:** Prevalence (%) of high blood pressure, underweight (BMI<18.5 kg/m^2^), overweight and/or obese (BMI≥23 kg/m^2^) and obese (BMI≥27.5 kg/m^2^) among the selected tribes (95% CI) with mean and standard deviation.

Tribes	High BP	Underweight	Overweight	Overweight and/or obese	BMI≥25 kg/m^2^; Mean (SD	Obese
		BMI<18.5 kg/m^2;^ Mean (SD)	BMI≥23 to <27.5 kg/m^2^ Mean (SD)	BMI≥23 kg/m^2^ Mean (SD)		BMI≥27.5 kg/m^2^; Mean (SD)
**Santal (West Bengal)**						
Prevalence	11.8 (8.4–16.5)	37.6 (31.5–43.6)	10.2 (6.8–14.9)	12.2 (8.7–16.9)	6.1 (3.7–9.9)	2.0 (0.01–0.1)
Mean (SD)	NA	17.0 (1.1)	24.7 (1.1)	25.4 (1.7)	26.7 (1.4)	28.5 (0.6)
**Kora**						
Prevalence	10.6 (7.3–15.2)	51.9 (45.5–58.3)	1.7 (0.7–4.3)	1.7 (0.7–4.3)	NOb.	NOb.
Mean (SD)	NA	16.4 (1.8)	23.8 (0.3)	23.8 (0.3)	NOb.	NOb.
**Oraon**						
Prevalence	16.5 (12.3–21.8)	49.6 (43.2–56.0)	6.8 (4.1–11.0)	7.6 (4.9–11.7)	2.5 (1.2–5.4)	0.9 (0.01–0.03)
Mean (SD)	NA	16.7 (1.3)	24.3(1.0)	24.9 (1.7)	26.9 (1.6)	28.7 (0.8)
**Santal (Odisha)**						
Prevalence	9.6 (6.5–14.0)	29.6 (23.8–35.4)	12.5 (8.7–17.5)	15.0 (11.0–20.1)	7.1 (4.5–11.1)	2.5 (0.01–0.1)
Mean (SD)	NA	17.2 (1.1)	24.5 (1.2)	25.3 (2.2)	27.1(2.0)	29.4 (1.1)
**Bhumij**						
Prevalence	14.7 (10.8–19.8)	28.2 (22.4–33.9)	15.6 (11.3–20.9)	17.7 (13.3–23.0)	8.0 (5.2–12.1)	2.1 (0.01–0.1)
Mean (SD)	NA	17.2 (1.0)	24. 7 (1.4)	25.4 (2.3)	27.3 (2.1)	29.9 (2.2)
**Bathudi**						
Prevalence	12.1 (8.5–16.8)	51.3 (44.9–57.6)	7.5 (4.6–11.8)	7.5 (4.8–11.5)	3.3 (1.7–6.4)	NOb.
Mean (SD)	NA	16.6 (1.5)	24.6 (1.0)	24.6(1.0)	25.6 (0.4)	NOb.
**Dhodia**						
Prevalence	12.9 (9.3–17.8)	28.8 (23.0–34.5)	20.9 (16.0–26.6)	23.8 (18.8–29.5)	11.3 (7.9–15.9)	2.9 (0.01–0.1)
Mean (SD)	NA	17 (1.2)	24.6 (1.3)	25.2 (2.4)	27.0 (2.0)	29.5 (2.0)
**Kukna**						
Prevalence	11.3 (7.9–15.9)	34.2 (28.2–40.2)	14.2 (10.2–19.5)	15.8 (11.8–21.0)	6.3 (3.8–10.1)	1.7 (0.01–0.1)
Mean (SD)	NA	17.1(1.1)	24.6 (1.1)	25.0 (1.2)	26.7 (1.5)	28.8 (0.8)
**Chaudhari**						
Prevalence	6.2 (3.8–10.0)	44.4 (38.1–50.7)	11.6 (8.0–16.5)	13.7 (9.9–18.6)	6.2 (3.8–10.0)	2.1 (0.01–0.1)
Mean (SD)	NA	16.8 (1.1)	24.5 (1.3)	25.0 (1.8)	26.7 (1.2)	27.9 (0.6)
**Total**						
Prevalence	11.7 (10.5–13.2)	39.4 (37.4–41.5)	11.2 (10.0–12.7)	12.8 (11.5–14.3)	5.7 (4.8–6.7)	1.6 (0.01–0.02)
Mean (SD)	NA	16.8 (1.3)	24.6 (1.2)	25.1 (1.9)	26.8 (1.7)	29.0 (1.6)

NA = Not applicable;

NOb. = Not observed in the data

The prevalence of hypertension, undernutrition, and overweight and/or obesity among men and women in each tribe is presented in Tables 3 and 4. The mean (with 95% CI) and SD for different categories of BMI are presented in Tables 3 and 4. The prevalence of high BP for men varied from 3.2% (95% CI 1.3–8.0) among Chaudharis to 13.3% (95% CI 8.4–20.6) among Kuknas, with a 9.2% prevalence (95% CI 7.6–11.2) among men. For women, it varied from 8.4% (95% CI 4.6–14.8) among Santals in Odisha to 23.4% (95% CI 16.8–31.6) among Oraons, with an overall prevalence of 14.0% (95% CI 12.1–16.2) among women. A high prevalence of undernutrition was observed among men and women of all the nine tribes. Women exhibited alarmingly high undernutrition prevalence (60% or more) among tribes, such as Koras, Oraons, and Bathudis.

**Table 3:** Prevalence (%) of high blood pressure, underweight (BMI<18.5 kg/m^2^), overweight and/or obese (BMI≥23 kg/m^2^), and obese (BMI≥27.5 kg/m^2^) among the men (95% CI) with mean and standard deviation.

Tribes	High BP	Underweight	Overweight	Overweight and/or obese	BMI≥25 kg/m^2^; Mean (SD	Obese
		BMI<18.5 kg/m^2;^ Mean (SD)	BMI≥23 to <27.5 kg/m^2^ Mean (SD)	BMI≥23 kg/m^2^ Mean (SD)		BMI≥27.5 kg/m^2^; Mean (SD)
**Santal (West Bengal)**						
Prevalence	9.8 (5.7–16.3)	30.1 (22.0–38.2)	12.2 (7.5–19.2)	12.2 (7.5–19.2)	4.9 (2.3–10.2)	NOb.
Mean (SD)	NA	17.2 (1.0)	24.9 (1.1)	24.9 (1.1)	26.0 (0.6)	NOb.
**Kora**						
Prevalence	7.9 (3.6–13.2)	41.2 (32.2–50.3)	1.8 (0.5–6.2)	1.8 (0.5–6.2)	NOb.	NOb.
Mean (SD)	NA	17.1 (1.2)	23.7 (0.4)	23.7 (0.4)	NOb.	NOb.
**Oraon**						
Prevalence	8.9 (4.9–15.7)	34.8 (26.0–43.6)	8.9 (4.6–16.2)	9.8 (5.6–16.7)	1.8 (0.5–6.3)	0.9 (0.01–0.1)
Mean (SD)	NA	17.0 (1.1)	24.2 (0.7)	24.6 (1.3)	27.0 (1.3)	27.9
**Santal (Odisha)**						
Prevalence	10.7 (6.4–17.5)	28.1 (20.1–36.1)	13.2 (8.0–20.9)	14.9 (9.6–22.3)	5.0 (2.3–10.4)	1.7 (0.5–5.8)
Mean (SD)	NA	17.2 (1.1)	24.3 (1.2)	24.9 (2.1)	27.3 (2.1)	29.8 (1.3)
**Bhumij**						
Prevalence	12.9 (8.0–20.2)	19.0 (11.8–26.1)	22.4 (15.4–31.3)	25.0 (18.0–33.6)	10.3 (6.0–17.2)	2.6 (0.01–0.1)
Mean (SD)	NA	17.2 (1.0)	24.7 (1.5)	25.1 (1.9)	27.0 (1.3)	28.7 (1.0)
**Bathudi**						
Prevalence	5.0 (2.3–10.6)	39.5 (30.7–48.3)	10.9 (6.5–17.8)	10.9 (6.5–17.8)	4.2 (1.8–9.5)	NOb.
Mean (SD)	NA	17.1 (1.2)	24.6 (1.0)	24.6 (1.0)	25.6 (0.5)	NOb.
**Dhodia**						
Prevalence	11.7 (7.1–18.6)	28.1 (20.1–36.1)	20.9 (14.2–29.4)	24.2 (17.4–32.6)	10.0 (5.8–16.7)	3.3 (0.01–0.1)
Mean (SD)	NA	16.9 (1.3)	24.2 (1.2)	24.8(1.9)	26.8 (1.6)	28.6 (0.6)
**Kukna**						
Prevalence	13.3 (8.4–20.6)	28.3 (25.5–42.5)	13.3 (8.2–21.4)	16.7 (11.1–24.4)	8.3 (4.6–14.7)	3.3 (0.01–0.1)
Mean (SD)	NA	17.0 (1.3)	24.6 (1.00)	25.4 (2.1)	26.9 (1.8)	28.8 (0.8)
**Chaudhari**						
Prevalence	3.2 (1.3–8.0)	40.0 (31.2–48.8)	12.8 (7.4–20.1)	16.1 (10.7–23.6)	8.1 (4.4–14.2)	3.3 (0.01–0.1)
Mean (SD)	NA	17.1 (1.0)	24.5 (1.3)	25.3 (1.9)	26.8 (1.3)	28.1 (0.6)
**Total**						
Prevalence	9.2 (7.6–11.2)	32.1 (29.3–34.9)	13.1 (11.1–15.3)	14.8 (12.8–17.0)	5.9 (4.7–7.5)	1.7 (0.01–0.03)
Mean (SD)	NA	17.1 (1.1)	24.5 (1.2)	25.0 (1.8)	26.7 (1.4)	28.6 (0.9)

NA = Not applicable;

NOb. = Not observed in the data

**Table 4:** Prevalence (%) of high blood pressure, underweight (BMI<18.5 kg/m^2^), overweight and/or obese (BMI≥23 kg/m^2^), and obese (BMI≥27.5 kg/m^2^) among the women (95% CI) with mean and standard deviation.

Tribes	High BP	Underweight	Overweight	Overweight and/or obese	BMI≥25 kg/m^2^; Mean (SD	Obese
		BMI<18.5 kg/m^2;^ Mean (SD)	BMI≥23 to <27.5 kg/m^2^; Mean (SD)	BMI≥23 kg/m^2^ Mean (SD)		BMI≥27.5 kg/m^2^; Mean (SD)
**Santal (West Bengal)**						
Prevalence	13.9 (8.2–20.2)	45.1 (36.3–53.9)	8.2 (4.2–14.9)	12.3 (7.6–19.3)	7.4 (3.9–13.4)	4.1 (0.02–0.1)
Mean (SD)	NA	16.9 (1.2)	24.5 (1.1)	25.8 (2.1)	27.2 (1.6)	28.4 (0.6)
**Kora**						
Prevalence	13.2 (8.3–20.4)	62.0 (53.3–70.6)	1.7 (0.5–5.8)	1.7 (0.5–5.8)	NOb.	NOb.
Mean (SD)	NA	15.9 (1.9)	23.9	23.9 (0.1)	NOb.	NOb.
**Oraon**						
Prevalence	23.4 (16.8–31.6)	62.9 (54.4–71.4)	4.8 (2.0–10.7)	5.6 (2.8–11.2)	3.2 (1.3–8.0)	0.8 (0.01–0.1)
Mean (SD)	NA	16.5 (1.3)	24.6 (1.3)	25.2 (2.1)	26.8 (1.8)	29.4
**Santal (Odisha)**						
Prevalence	8.4 (4.6–14.8)	31.1 (22.8–39.4)	11.8 (6.8–19.3)	15.1 (9.8–22.7)	9.2 (5.2–15.8)	3.4 (1.3–8.3)
Mean (SD)	NA	17.2 (1.0)	24.7 (1.1)	25.7 (2.5)	26.9 (2.0)	29.2 (1.3)
**Bhumij**						
Prevalence	16.4 (10.9–24.0)	37.0 (28.3–45.5)	9.0 (4.8–15.9)	10.7 (6.3–17.4)	5.7 (2.8–11.4)	1.6 (0.01–0.1)
Mean (SD)	NA	17.2 (0.9)	24. 9 (1.4)	25.9 (3.0)	27.7 (3.1)	31.8 (2.4)
**Bathudi**						
Prevalence	19.0 (13.0–26.9)	62.8 (54.2–71.4)	4.1 (1.8–9.3)	4.1 (1.8–9.3)	2.5 (0.9–7.0)	NOb.
Mean (SD)	NA	16.3 (1.6)	24.7 (1.2)	24.7 (1.8)	25.6 (0.3)	NOb.
**Dhodia**						
Prevalence	14.2 (9.0–21.5)	29.2 (21.0–37.3)	20.8 (14.2–29.4)	23.3 (16.7–31.7)	12.5 (7.7–19.6)	2.5 (0.01–0.1)
Mean (SD)	NA	17.1 (1.0)	25.0 (1.3)	24.8 (2.4)	27.1 (2.3)	30.8 (2.5)
**Kukna**						
Prevalence	9.2 (5.2–15.7)	40.0 (31.2–48.8)	15.0 (9.7–22.5)	15.0 (9.7–22.5)	4.2 (1.8–9.4)	NOb.
Mean (SD)	NA	17.2 (0.9)	24.6 (1.2)	24.6 (1.2)	26.3 (0.4)	NOb.
**Chaudhari**						
Prevalence	9.1 (5.2–15.6)	48.8 (39.9–57.7)	9.9 (5.5–17.0)	10.7 (6.4–17.5)	4.1 (1.8–9.3)	0.8 (0.01–0.1)
Mean (SD)	NA	16.5 (1.2)	24.3 (1.4)	24.6 (1.7)	26.4 (1.1)	27.6
**Total**						
Prevalence	14.0 (12.1–16.2)	46.6 (43.7–49.6)	9.5 (7.8–11.4)	10.9 (9.2–12.9)	5.4 (4.2–7.0)	1.5 (0.01–0.02)
Mean (SD)	NA	16.6 (1.4)	24.7 (1.2)	25.3 (2.1)	26.9 (2.0)	29.5 (2.0)

NA = Not applicable;

NOb. = Not observed in the data

A 14.8% prevalence of overweight and/or obesity (95% CI 12.8–17.0) was observed among men, with1.69% (95% CI 0.01–0.03) in the obese (BMI≥27.5 kg/m^2^) category. Five of the nine tribes exhibited approximately 15% prevalence or more of a BMI≥23 kg/m^2^ and 5% or more of a BMI≥25 kg/m^2^. The prevalence of a BMI≥23 kg/m^2^ among women was 10.9% (95% CI 9.2–12.9), with 1.5% (95% CI 0.01–0.02) in the BMI≥27.5 kg/m^2^ (obese) category. Among women, six of the nine tribes displayed more than 10% prevalence of a BMI≥23 kg/m^2^, with Dhodia women displaying the highest frequency of 23.3%. Women from four of the nine tribes exhibited more than 5% prevalence of a BMI≥25 kg/m^2^, with the highest of 12.5% among Dhodias.

**Fig 5:**
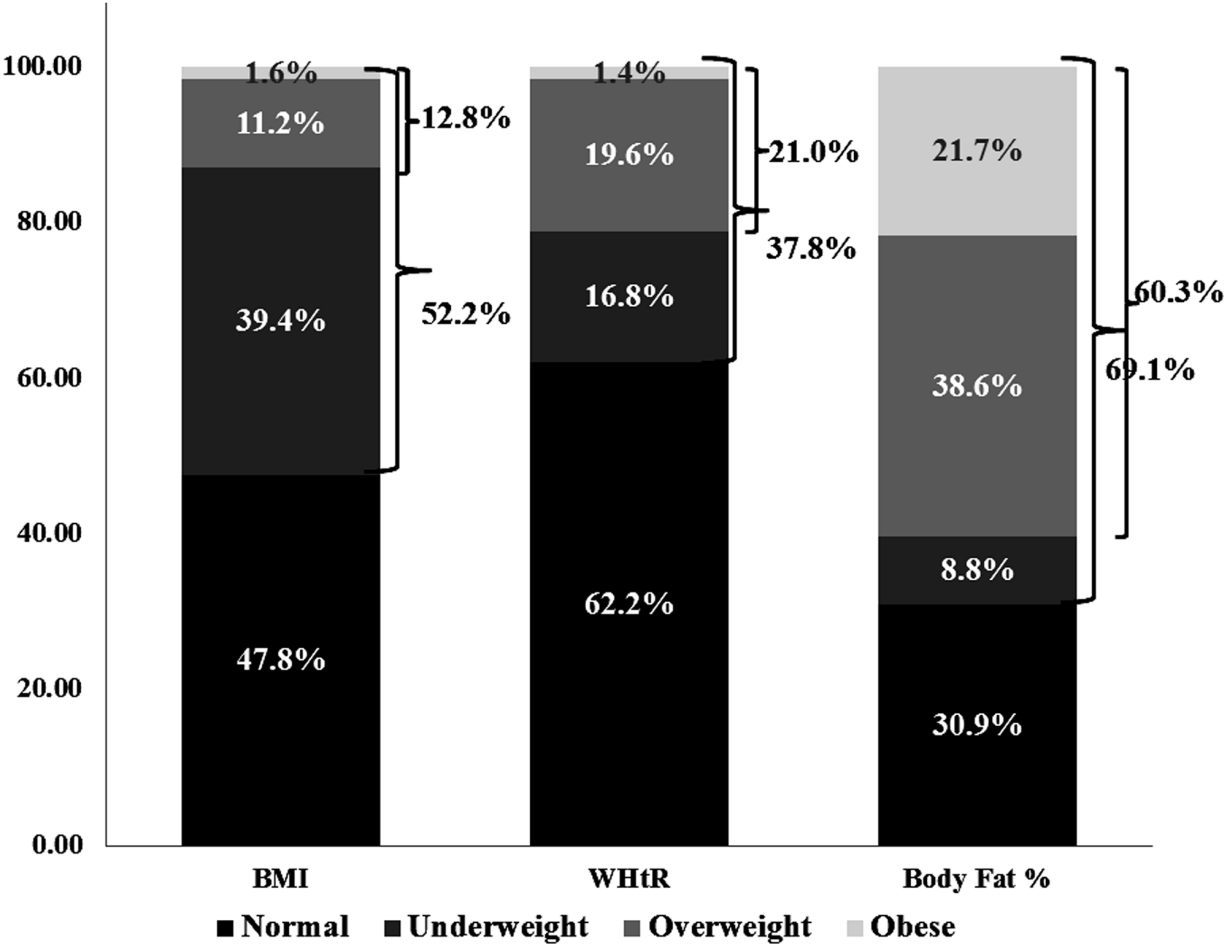
Double burden of malnutrition among the nine tribes. **Note:** Each stacked bar denotes the double burden of overweight and obesity as well as undernutrition based on BMI, WHtR and body fat percentage.

The double burden of undernutrition and obesity among men and women is given in Fig 5. A 52.2%, 37.8%, and 69.1% risk of the double burden was observed when considering BMI, WHtR, and body fat percentage as the measuring index, respectively. Although the prevalence of double burden is variedly explained by the three different parameters, they indicate an increasing trend of the coexistence of undernutrition and obesity along with hypertension among Indian tribal populations.

**Fig 6:**
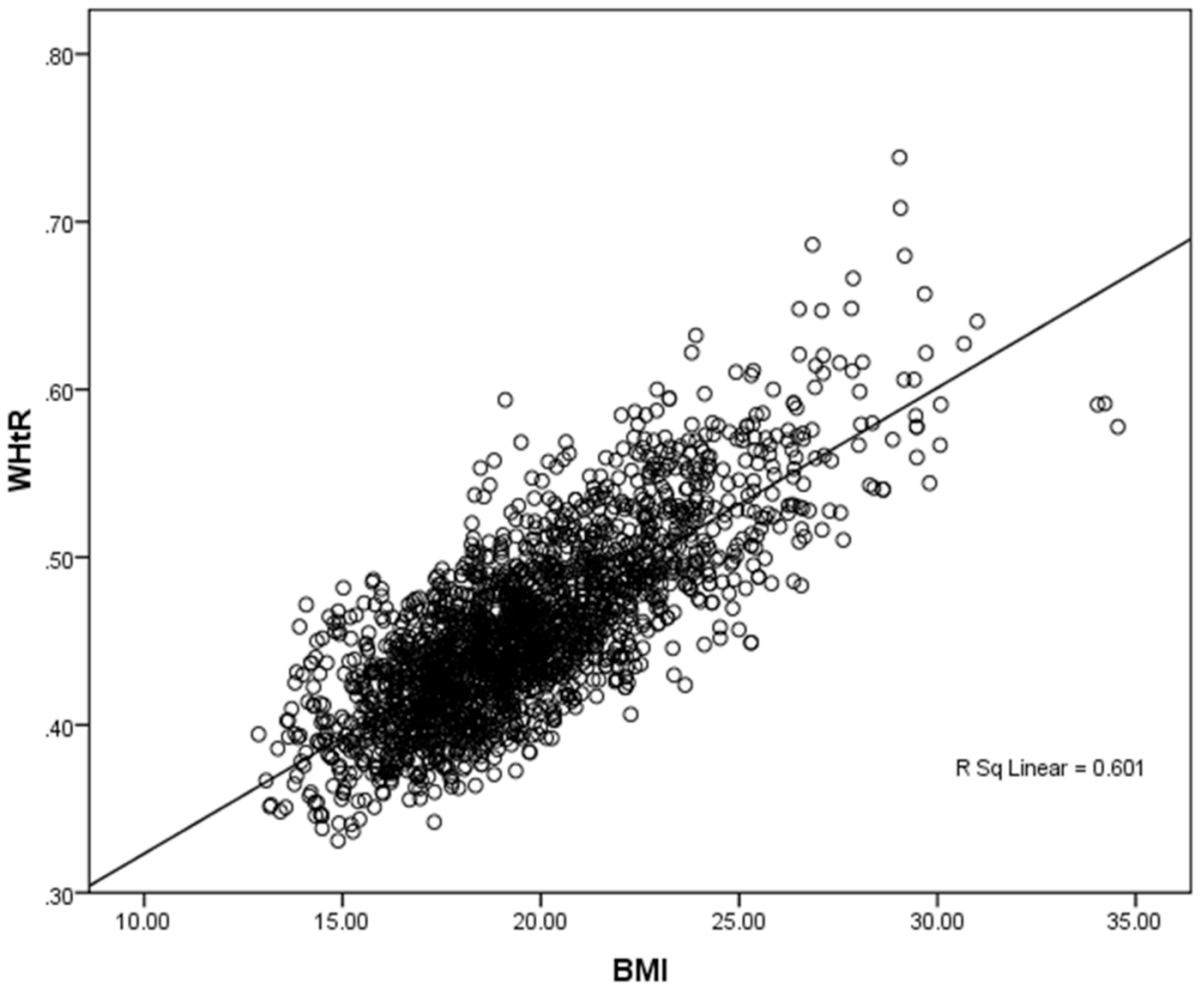
Scatter plot of interaction between BMI and WHtR across the overall study population.

Furthermore, Fig 6 illustrates the interaction between BMI and WHtR in the overall population. While both variables comprehensively explain the substantial presence of nutritional and CVD risks in the population, the comparison simultaneously demonstrates both variables as significant predictors of metabolic risks in Indian tribal populations.

## Discussion

The present study provides crucial insights regarding the tribal health in India. An alarming -fold increase in overweight and/or obesity (12.81%) was observed, compared to the 2–3% increase reported previously [9], with an average adult undernutrition prevalence as high as 40% and hypertension prevalence of more than 10%, which requires immediate attention. High accumulated body fat percentage among Indian tribes is another area of concern reported by our findings. It is the first large-scale comprehensive study on the prevalence of undernutrition, overweight or obesity, and hypertension among the highly disadvantaged Indian tribal population. The findings on the three considered weight variables, such as BMI, WHtR, and body fat percentage, reveal that more than half of the population suffers from the double burden of malnutrition, whereas one among five individuals from the double burden bracket is hypertensive. These findings clearly demonstrate that the health status of Indian tribes is a cause of concern, which contradicts the traditional wisdom that these population groups are free from non-communicable lifestyle diseases.

The findings revealed a high prevalence of hypertension among the studied Indian tribes, with a high prevalence of undernutrition and overweight among women and men respectively. The prevalence of obesity was similar among men and women. The increased prevalence of overweight along with high undernutrition and hypertension indicated that women are at a higher risk of CVD-related vulnerabilities. This further implies a strong gender inequality with respect to food security [49].

Recent evidence indicated that extensive urbanization is strong risks factor for CVDs a [50]. Major tribal-dominated states, such as Maharashtra, West Bengal, Andhra Pradesh, Gujarat, and Madhya Pradesh have experienced a high rate of urbanization, with more than 20–30% increase during 2001–2011 (census year) [12]. The population growth rate increased consistently (at 30%) in the urban areas and sharply declined in rural areas, whereas the rural literacy rate increased remarkably compared to that of urban literacy rate [51]. Thus, urban populations experiencing a high population growth have exhibited a broad shift in life style changes including the structure of diet and physical activities [52–55]. On the other hand, rural and tribal populations are increasingly gaining better access to information regarding various sources of food products and entertainment in the neighborhood, which plays a pivotal role in their availability, acceptability, and (in certain cases) affordability of lifestyle changes, ranging from life style changes, to food habits[56–59]. Similar trends are further substantiated in the present study.

Among medium and low human development index countries, there is an increasing tendency in the society towards globalized food markets while various processed and packaged foods are increasingly being considered popular and a prestige symbol [60, 61]. The effects of such nutritional transition weighs heavily on the lower socio-economic sections in the society [62]. In his study, Hawkes revealed that this globalization-driven nutritional transition, with drastic changes in food habits, further deepens the status of nutritional inequality among the rich and poor; the poor are the worst affected by a cultural convergence towards low quality diets (such as inexpensive vegetable oil and trans-fat) [63] Therefore providing mere nutritional literacy or dietary information or accessibility is not sufficient for better nutrition, a better food policy is required to improve the affordability of this economically deprived section to enhance their access to better quality of foods [64].

The Indian population census does not provide any data on tribal migration; however, we explored the role of migration in the socio-cultural and other changes among the studied tribes. Noteworthy trends of migration among the nine tribes was absent except limited intra-state migrations and few seasonal inter-state migrations. However the present study findings highlighted the high rate of changes in the living practices of these tribes. Several urban and market centers have developed in and around the tribal regions, which have considerable influence the socio-cultural lifestyle and habits of the nearby inhabiting tribes. A glimpse of this adaptation and shift from indigenous traditions to an urban lifestyle was considerably visible during fieldwork. For example, among the Santals of Odisha, the traditional community dances, which were common practices in every village during festivals, are now being replaced by television shows of movies and music. Among the Chaudharis of Gujarat, a 70-year old lady elaborated her experience saying, “Previously, the work pressure in family was less as life was simple and the expectations were less. We had time to relax, merrymaking, dance, and community gathering. But in present day conditions, I have to work throughout the day at this age and ironically I am undergoing hypertension treatment as well. It is not that my family wants me to work; rather I have to work, so that I can support my children to fulfill their wish to live a modern life. But, I hardly enjoy all these activities.”

Ghurye (1963) stated that industrial growth was not the only reason for the urbanization of rural India; it began within the rural areas itself. Taking references from Sanskrit texts and documents, he illustrated the growth of urban centers in rural hinterlands as an outcome of the need for markets [2]. Gautam (1977) in his seminal work in Santal Pargana reported similar observations regarding the development of ‘market centers’ in Santal-populated areas which evidently influenced tribal lives [3]. These ‘urban or market centers’ consistently became a place for social interaction among the tribes. Thus, such external influences on lifestyle substantially contributed to the changing health status of tribes over time.

The second important observation was that the tribal families were mostly covered by below poverty line (BPL) schemes of the state and central governments. They are supplied with rice, wheat, and sugar at highly subsidized rates under these schemes [65]. Such provisions have increased their dependency on the public food assistance program. Furthermore, their previous practices of food collection from forests are now restricted because of various forest protection acts [66, 67], which led to restricted diversity in their food basket. Azadbakht and Esmaillzadeh (2011) demonstrated that restricted diversity in diet is strongly associated with obesity and other co-morbidity factors among Iranian women [68]. Evidences supporting the association between low-diversity in diet and nutritional deficiencies were revealed in another recent study [69]. Therefore, the shift from a traditionally enjoyed food sovereignty to a shrinking food basket with a high dependency on supplied food provided by public distribution systems among Indian tribal populations may be an important cause of undernutrition and obesity among these tribes. Similar findings among other vulnerable groups explain that the high prevalence of obesity is an outcome of their suffering from severe cultural and economic disruptions with prolonged food insecurity, followed by a rapid transition to more refined foods [70, 71]. Pima Amerindians, who were confined to reservations by a growing dependence on food assistance for generations while undergoing a rapid transition from a nomadic to a settled lifestyle, exhibited high levels of obesity and type 2 diabetes during later condition [72]. An increased possibility of developing obesity was observed among individuals with undernutrition in early life followed by an exposure to refined, carbohydrate-rich diets [71, 73]. A 3-to 4-fold increase in overweight and a significant prevalence of obesity in the study population supports these findings.

High undernutrition is a consistent trend among Indian tribal children and adults [74]. Severe wasting and stunting has been reported among Indian tribal children and adolescents. [75–80, 28]. Our findings reported a similar trend in post-adolescent life with high nutritional deficiencies and food insecurity among the studied young adults and elderly groups. Evidence from shantytowns in Brazil suggested that stunted children are at a higher risk of obesity in later life because of an impaired fat oxidation capacity (a risk factor for obesity) [81], whereas associated factors of undernutrition in early life increase the susceptibility to overweight, later in life [82, 83].

Previous studies have revealed hypertension as a critical adverse health outcome with undernutrition and obesity [84–85]. The findings of the present study revealed an increased prevalence of hypertension among undernourished and obese men as well as women. Evidences from Brazil and other studies show that stunting or chronic undernutrition increases the risk of obesity and hypertension, irrespective of age with an increased vulnerability among socio-economically marginalized groups [83]. Women were at a higher risk of hypertension than men [83], which was also observed in our study.

Therefore, the present study reveals several major findings. It demonstrated that a lack of quality and diversity of diet in a regular food basket and food assistance program can cause food insecurity [49, 71].

Urbanization and its triggered lifestyle changes, marginalization in the broad socioeconomic context, and cultural shift are crucially associated and suggestive factors of a non-communicable disease burden and nutritional extremes among Indian tribes. Finally the study reports for the first time, a comprehensive picture of adverse health conditions among Indian tribal populations that are largely disadvantaged groups of India. Therefore, a systematic and comprehensive health policy along with timely intervention programs is strongly warranted.
